# Feasibility trial of a film-based educational intervention for increasing boys’ and girls’ intentions to avoid teenage pregnancy: Study protocol^☆^

**DOI:** 10.1016/j.ijer.2014.08.003

**Published:** 2014

**Authors:** Maria Lohan, Áine Aventin, Lisa Maguire, Mike Clarke, Mark Linden, Lisa McDaid

**Affiliations:** a School of Nursing and Midwifery, Queen's University Belfast, Northern Ireland/Scotland, United Kingdom; b Centre for Public Health, Queen's University Belfast, United Kingdom; c MRC/CSO Social & Public Health Sciences Unit, University of Glasgow, United Kingdom

**Keywords:** Teenage pregnancy, Sex education, Sexual health promotion, Intervention, Feasibility trial

## Abstract

The World Health Organisation, amongst others, recognises that adolescent men have a vital yet neglected role in reducing teenage pregnancies and that there is a pressing need for educational interventions designed especially for them. This study seeks to fill this gap by determining the feasibility of conducting an effectiveness trial of the *If I Were Jack* intervention in post-primary schools. This 4-week intervention aims to increase teenagers’ intentions to avoid unintended pregnancy and addresses gender inequalities in sex education by explicitly focusing on young men. A cluster randomised feasibility trial with embedded process evaluation will determine: recruitment, participation and retention rates; quality of implementation; acceptability and feasibility of the intervention and trial procedures; and costs.

## Highlights

• We report the protocol of a feasibility trial for a school-based sex education intervention.
• The intervention challenges the gender-bias in sex education by especially targeting boys.
• The interactive online intervention prompts adolescents to think about teenage pregnancy.
• The cluster randomised controlled trial includes a process evaluation and health economics analysis.
• This trial will test the feasibility of a larger trial to test for intervention effectiveness.

## 1 Introduction

Teenage pregnancy remains a world-wide public health concern with rates in the US and UK among the highest in high-income countries (Finer and Zolna, 2011, Lawlor and Shaw, 2004). While teenage pregnancies have been gradually decreasing over the past decade, recent figures suggest pregnancy rates as high as 54.6 per 1000 women under 20 in the UK (ONS, 2012) and 67.8 per 1000 in the US (Kost & Henshaw, 2012). As many as half of these pregnancies end in legal abortion, reflecting the potentially unintended or unwanted nature of these conceptions (Kost and Henshaw, 2012, ONS, 2012). Although the life course for teenaged parents is not universally negative (Bonell, 2004, Duncan et al., 2010), the social disadvantage and exclusion that are linked to *unintended* teenage pregnancy are considered problematic (Harden, Brunton, Fletcher, & Oakley, 2009). Unintended adolescent pregnancy can lead to considerable adverse health problems for teenagers and their infants as well as generating emotional, social and economic costs for adolescents, their families and society (Beers & Hollo, 2009).

While unintended teenage pregnancy is a complex phenomenon that cannot be prevented through Relationship and Sexuality Education (RSE) alone (DiCenso et al., 2002, Elliott et al., 2013, Fullerton, 2004, Henderson et al., 2007, Shepherd et al., 2010, Silva, 2002, Stephenson et al., 2003, Wight et al., 2002), it is recognised that high quality RSE provides teenagers with a solid knowledge base on which to make informed decisions about their sexual behaviour, as well as being a vital aspect of improving holistic sexual health and wellbeing (Downing et al., 2006, Ellis and Grey, 2004, Ingham and Hirst, 2010, Oringanje et al., 2009, Swann et al., 2003). Reflecting the importance of RSE in the UK, the governments of Northern Ireland (NI), England and Scotland all emphasise the need to achieve a reduction of teenage pregnancy rates via the implementation of RSE in schools and see this as a key objective in their sexual health policies (Department of Health, 2013, DHSSPS, 2008, The Scottish Government, 2011).

In recent times, there has been recognition that teenage men have a vital yet neglected role in reducing teenage pregnancies (Alan Guttmacher Institute, 2002, Lindberg and Kost, 2014, Marsiglio, 2006, Saewyc, 2012, Smith et al., 2005, Swann et al., 2003). When young men do receive RSE concerning pregnancy it is often via programmes and interventions that are directed towards girls and which ignore the fact that males and females are affected differently by gender norms and values relating to pregnancy (World Health Organisation, 2011). Thus, internationally, researchers and policy makers have called for targeted and scientifically evaluated RSE interventions which meet the sexual health needs of young men (Juszczak and Ammerman, 2011, Saewyc, 2012, World Health Organisation, 2011).

While some behavioural programmes targeting adolescent sexual risk-taking behaviour have demonstrated only modest success (DiCenso et al., 2002, Shepherd et al., 2010), systematic reviews have identified the characteristics of effective RSE programmes which might help optimise their potential impact on sexual risk-taking behaviours (Bailey et al., 2010, Guse et al., 2012, Kirby et al., 2006, Kirby, 2007, Kirby, 2002, Noar et al., 2010, Robin et al., 2004, Shepherd et al., 2010). These include the use of theoretically-based interventions targeting sexual and psychosocial mediating variables such as knowledge, attitudes, self-efficacy, intentions, perceptions of risk, and perceptions of peer norms which are theoretically linked to sexual behaviour change (Ajzen and Madden, 1986, Cane et al., 2012, Michie et al., 2013, Rivis et al., 2009); the use of culturally-sensitive and gender-specific interventions (Marsiglio, 2006, Ries and Sonenstein, 2006); the use of interactive modalities which promote personal identification with the educational issues and engagement of young people (Bailey et al., 2010, Guse et al., 2012); the use of skills-building components (Oringanje et al., 2009, Wight et al., 1998); the involvement of parents in the RSE process (Grossman, Frye, Charmaraman, & Erkut, 2013); and the facilitation of linkages with sexual health support services (Coyle et al., 1999).

The *If I Were Jack* intervention represents an innovative combination of these different components and is therefore predicted to decrease young people's sexual risk-taking behaviour in relation to avoiding teenage pregnancy. The aim of the study described in this protocol is to explore the feasibility of determining, in a cluster randomised trial, whether this combination of components can effectively increase boys’ and girls’ intentions to avoid an unintended teenage pregnancy.

## 2 Methodology

### 2.1 Background to the study

**Fig. 1:**
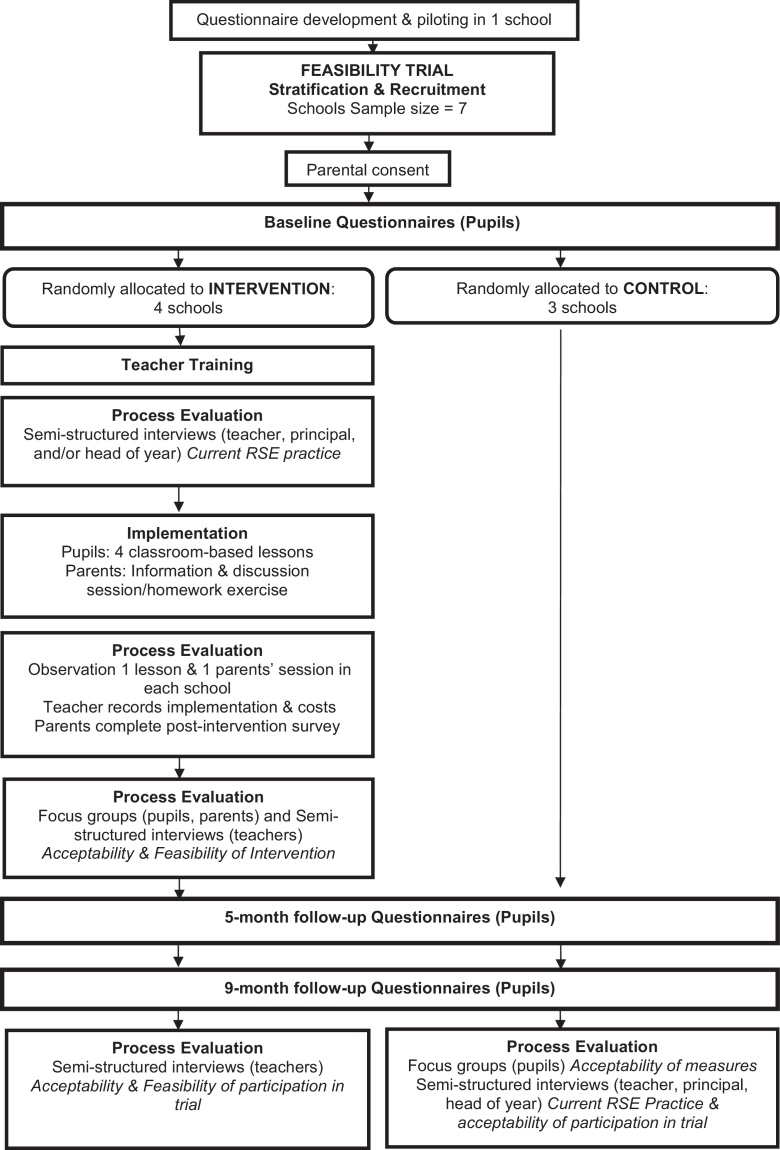
Jack Feasibility Trial Project Flowchart.

The study is a cluster randomised feasibility trial with embedded process and cost-effectiveness evaluations. The project began in May 2014 and will run for two years (see project flowchart, Fig. 1). Following the UK Medical Research Council's *Guidelines for the Development and Evaluation of Complex Interventions* (Medical Research Council, 2008), the current phase of research was preceded by focused programme development (Aventin, Lohan, O’Halloran, & Henderson, 2014) which involved identification of the relevant evidence base, development of a theoretical understanding of the phenomenon of unintended teenage pregnancy in relation to young men, and exploratory mixed methods research examining the acceptability of the *If I Were Jack* interactive film among young men, teachers and RSE specialists (Lohan et al., 2011, Lohan et al., 2012).

### 2.2 Intervention

Informed by research on adolescent men's attitudes towards unintended pregnancy and the best available evidence on the components of effective RSE interventions, the intervention was designed to fit within the NI RSE curriculum by a team of researchers at Queen's University Belfast working with experts from the Department of Health, Social Services and Public Safety NI, the Public Health Agency NI, and the Council for the Curriculum, Examinations and Assessment in NI, as well as RSE specialists, teachers, parents and young people. Although produced in NI, the intervention will have strong cultural resonance for Ireland and the UK as a whole and could be adapted for use internationally.

This 4-week programme is composed of a number of elements (Table 1):

i. *If I Were Jack* interactive video drama (IVD) which tells the fictional story of 16-year old Jack who has just found out that his girlfriend is pregnant. Seated at individual computers, pupils are immersed in Jack's story and are asked to consider how they would feel and what they would do if they were Jack;
ii. Classroom materials for teachers containing four detailed lesson plans with specific classroom-based and homework activities which include group discussions, role-plays, worksheets, and a parent-pupil exercise;
iii. 60-min training session delivered by a researcher to teachers wishing to implement the intervention detailing the components of the intervention and its delivery and highlighting the research procedures;
iv. 60-min information and discussion session for parents/guardians delivered by RSE teachers during the first week of the implementation period (prior to the parent/guardian homework activity; and
v. Detailed information brochures, factsheets and a dedicated website (www.qub.ac.uk/IfIWereJack) about the intervention and unintended teenage pregnancy in general for schools, teachers, teacher trainers, young people and parents.

**Table 1:** Psychosocial and behavioural components of the *If I Were Jack* intervention.

Component	Aim	Measurement
Knowledge	Increase knowledge about: ways of avoiding unintended pregnancy; roles and responsibilities of young men in relation to unintended pregnancy; possible negative relational, social, emotional and financial consequences of unintended pregnancy; and sources of information and support for unintended pregnancy	Individual assessment. Total score on 10 knowledge items. Selected items from the Mathtech Knowledge Inventory (Kirby, 1984); and SKATA (Fullard et al., 2005)

Communication skills	Increase skills communicating with parents and peers about avoiding unintended pregnancy	Comfort Communicating about Avoiding Unintended Teenage Pregnancy Scale(parents, peers and professionals) using adapted items from the Mathtech Behaviour Inventory (Kirby, 1984)

Attitudes about unintended pregnancy	Increase anticipated regret about the consequences of unintended pregnancy on current life and future goals	Items from the newly developed Intention to Avoid Unintended Teenage Pregnancy Scale

Social influences	Increase awareness of peer norms, stereotypical gender norms and parental attitudes and beliefs about teenage pregnancy *Gender norms:* increase perception that both men and women have roles and responsibilities in avoiding and dealing with the consequences of unintended pregnancy *Peer norms:* increase perception that most peers are not sexually active and use contraception when they are *Parental values & beliefs:* increase awareness of parental attitudes and beliefs about unintended pregnancy	*Male Role Gender norms:* Male Role Attitudes Scale (Pleck et al., 1993) and knowledge items relating to responsibility for avoiding pregnancy. *Peer norms:* Knowledge items about sexual behaviour/contraceptive use among peers and sexual socialisation instrument (peer sub-scale) (Lottes & Kuriloff, 1994) *Parental values & beliefs:* Sexual socialisation instrument (parent sub-scale) (Lottes & Kuriloff, 1994)

Beliefs about capabilities	Increase perceived behavioural control to avoid unintended pregnancy (say no to sex or obtain and use contraception correctly) and increase self-efficacy to communicate about avoiding unintended pregnancy with parents, peers & professionals	Sexual self-efficacy scale using an adapted version of the Sexual Self-Efficacy Scale (Rosenthal, Moore, & Flynn, 1991)

Intentions	Increase strength of intention to avoid unplanned teenage pregnancy	Intentions to avoid unintended teenage pregnancy scale (constructed by research team)

Sexual behaviour	Abstinence from sexual intercourse (delay initiation of sex or return to abstinence) or avoidance of unprotected sexual intercourse (consistent correct use of contraception)	Sexual behaviour items (ever had sexual intercourse; frequency of sexual intercourse; contraception use ever/at last intercourse). Items adapted from previous sexual health surveys (Carswell et al., 2012, Elliott et al., 2010).

Pregnancy	Avoidance of unintended pregnancy	Ever pregnant

### 2.3 Control group

Pupils in the control group of the study will not receive the *If I Were Jack* intervention and will continue with normal RSE practice. We will determine resistance to assignment to the control group by recording refusal to participate or difficulties with retention at follow-up. If we experience difficulties in this regard, we will consider alternative designs for the main trial.

#### 2.3.1 Theoretical framework

The theoretical basis of the *If I Were Jack* intervention has been developed from the research team's systematic review of the literature on adolescent men's attitudes and decision-making in relation to an unintended pregnancy (Lohan, Cruise, O‘Halloran, Alderdice, & Hyde, 2010). The review indicated a number of potential influences on young men's attitudes to adolescent pregnancy and pregnancy outcomes including: social class; religiosity; gender identity (some studies found links between traditionally masculine ideals and perceptions that sexual experience validated a masculine identity); attitudes and subjective norms regarding how significant others (such as partners, friends and parents) would expect them to behave in such a situation; and the idealisation of pregnancy and parenthood. This theoretical framework combines with the well-established Theory of Planned Behaviour (Ajzen, 1988, Ajzen and Madden, 1986) and more recent enhancements of the model such as the Integrated Behavioural Model (Montano & Kasprzyk, 2008) and behaviour change frameworks (Cane et al., 2012), all of which underpin the content of the *If I Were Jack* intervention. The Theory of Planned Behaviour (TPB) suggests that behaviour is influenced by intention, which is in turn influenced by a range of psychosocial mechanisms such as attitudes and beliefs, perceived norms and perceptions of personal control over the behaviour. TPB has been supported by extensive research (for a meta-analysis of this research, see (Albarracin, Johnson, Fishbein, & Muellerleile, 2001) while others have extended the model to include knowledge, skills building and anticipated affect (i.e. the prospect of feeling positive or negative emotions after performing a behaviour) (Abraham et al., 2004, Montano and Kasprzyk, 2008, Rivis et al., 2009). It has also been extended to promote an awareness of relevant broader socio-cultural factors (Abraham et al., 2004) which, in the case of the *If I were Jack* intervention, were drawn from our systematic review on adolescent men and unintended pregnancy (Lohan et al., 2010). It is therefore hypothesised that the *If I Were Jack* intervention will have its effect via its impact on knowledge, skills, beliefs about consequences and capabilities, and increased awareness of social influences which will, in turn, have their impact on behaviour via pathways through a young person's intention to avoid sexual risk taking behaviour.

### 2.4 Outcome measures and assessment instruments

Key outcomes are the quality of intervention implementation and recruitment and retention of participants. The study will also pilot the feasibility and acceptability to participants of providing demographic data and answering sexual behaviour questions measuring the proposed primary and secondary outcomes for a future, larger phase III trial. Drawing upon the United Kingdom Medical Research Council Guidelines (MRC, 2008), a phase III study is a randomised controlled trial in which the sample size is statistically powered to capture the effectiveness of an intervention.

In a phase III trial, a reduction in unintended adolescent pregnancy rates would be the ideal primary outcome measure. However, the sample size would need to be large in order to detect an important change in this. We will therefore use surrogate measures associated with reductions in unintended pregnancy: abstinence from sex or avoidance of unprotected sex (2 years past baseline). In the feasibility trial, we will pilot the feasibility and acceptability of collecting these data from pupils at five and nine-month follow-up.

Secondary outcomes in a phase III trial would be six-month impacts on knowledge, attitudes, skills and intentions relating to avoiding teenage pregnancy. These short-term impacts are hypothesised to lead to increased intention to avoid teenage pregnancy. In this feasibility trial, we will collect data from pupils using items developed specifically for use in the study as well as items from a number of adapted standardised measures to provide broad estimates of effect sizes and the feasibility and acceptability of the questions. The measures were chosen because the constructs they measure map closely to the theoretical framework underpinning the intervention. As part of the study, we will develop a measure of intentions to avoid unplanned pregnancy. We will investigate the association between intentions at baseline and behaviour (abstinence or avoidance of unprotected sex) at follow-up. While regression analyses would be used in the main trial to model the impact of intentions on behaviour, the feasibility study is not powered to detect statistical differences and we will present descriptive statistics relating to the primary outcomes.

**Knowledge** will be measured with items which replicate or have been adapted from similar items in existing validated sexual knowledge scales [Sexual Knowledge & Attitudes Test for Adolescents (Fullard, Johnston, & Lief, 2005); Mathtech Knowledge Inventory (Kirby, 1984)] and questionnaires used in previous sexual health studies with adolescents (Carswell et al., 2012, Elliott et al., 2010).

**Communication skills** will be assessed using adapted versions of the comfort in communicating with peers and parents about sex and birth control sub-scales of the Mathtech Behaviour Inventory (Kirby, 1984). We have adapted the sex sub-scale so that it refers to comfort communicating about avoiding pregnancy and added a new item which assesses comfort in communicating with professionals about unintended pregnancy and contraception.

**Attitudes towards unintended pregnancy** will be measured using items from a newly developed measure of intention to avoid unintended pregnancy (see below).

**Awareness of stereotypical gender norms** will be assessed using the Male Role Attitudes Scale (Pleck, Sonenstein, & Ku, 1993) and attitude items relating to male/female responsibility for avoiding unintended pregnancy.

**Awareness of peer norms and parental values and beliefs** relating to avoiding unintended pregnancy will be assessed using the peer and parent sub-scales of the Sexual Socialisation Instrument (Lottes & Kuriloff, 1994).

**Beliefs about capabilities in avoiding unintended pregnancy** and communicating about avoiding unintended pregnancy will be measured using an adapted version of the Sexual Self-Efficacy Scale (Rosenthal, Moore, & Flynn, 1991).

A measure of **intention to avoid teenage pregnancy** will be developed as part of the study.

**Avoidance of sexual risk-taking behaviour** (abstinence from sexual intercourse or avoidance of unprotected sex) will be assessed using eight items adapted from previous sexual health surveys (Carswell et al., 2012, Elliott et al., 2010). Incidence of pregnancy will be recorded in order to establish **pregnancy outcomes.**

### 2.5 Questionnaire development & piloting

Before data collection we will develop the questionnaire, based on the measures described above, ensuring that it takes no longer than 30 min to complete (the average length of RSE classes in NI).

In June 2014, we will ask 40 Year 11 pupils (aged 14–16 years) in a school that is not taking part in the trial to complete an online version of the questionnaire. We will solicit qualitative comments on items and use statistical analyses to reduce the length and select items relating to key concepts. This piloting will determine whether data generated from the questionnaire is valid and reliable. Content validity has to some degree already been established through discussion and selection of items within the research team. This will be demonstrated further through the participants’ ability to complete the questionnaire in a timely and consistent fashion. We will use Cronbach's alpha to test whether the individual scales contained within the questionnaire possess internal consistency and reliability when used with the target population. A subset of participants will take part in two focus group discussions to identify any procedural difficulties in completing the questionnaire. Discussions will focus on completion times, language, usability and comfort with the subject matter. If participants indicate dissatisfaction with certain aspects of the questionnaire, these will be reviewed by the research team, who may decide to alter or remove the items.

The resulting questionnaire will then be used in the feasibility study. During the feasibility study we will also ask a sample of pupils in the control group to participate in cognitive interviews regarding the acceptability and feasibility of the questions. Using responses from the entire sample, scales will be assessed for validity by means of exploratory principle components analysis and for internal consistency by Cronbach's alpha.

### 2.6 Sample

Seven post-primary schools in NI will be recruited to the feasibility trial. Eligible schools will include those that currently have a Year 11 group (i.e. adolescents aged 14–16 years). There are 216 such schools in NI in 2012/13. Schools will be excluded if they are defined as hospital, independent or Irish language schools or have less than 30 pupils in Year 11. The study sample will also be stratified to reflect the main categories of schools. In NI schools are categorised as ‘secondary’ and ‘grammar’ with the latter using academic ability to select pupils. Various management structures exist with ‘controlled’ schools managed by one of five education and library boards and ‘voluntary’ schools managed by a board of trustees (usually local churches). The Catholic Church manages a significant number of voluntary (‘maintained’) schools. Although religion is not a criterion for attendance, most pupils at controlled schools are from Protestant denominations and most of those attending maintained schools are Catholic. There are also a number of ‘integrated’ schools, which aim to provide a religiously mixed environment. In order to capture diversity and reflecting the organisation of education in the special circumstances of NI, recruited schools will consist of the following: 1 controlled integrated; 1 controlled grammar; 2 voluntary Catholic grammar; 2 secondary schools in deprived areas; and 1 voluntary ‘other’ management.

Potentially eligible schools will be identified from those attending RSE teacher training (as provided by the Public Health Agency of NI). This training session will introduce the intervention and provide an overview of the research. RSE teachers in attendance will be asked to indicate if they are interested in receiving more information about the research. The schools that we will approach in the first instance will be selected on the basis of 1) their expressed interest and 2) their fit with our recruitment criteria. Schools which decline to participate will be replaced by the next listed school from the list of all eligible schools stratified by school management type and deprivation.

The target population is adolescents aged 14–16 in Year 11. The total sample size of seven schools is likely to provide access to approximately 630 pupils. This is based on the average size of year 11 groups in schools in 2011/12 (mean = 114; median = 113) (DOE, 2012) and allows for an 80% consent rate (Henderson et al., 2007).

### 2.7 Randomisation procedures

Schools will be the unit of randomisation, with four randomly allocated to the intervention group and three to the control group. After baseline data collection, the two sets of pairs (Catholic grammar and deprived secondary) will be randomised as pairs to ensure that one of each type is in the intervention arm. Simple random selection will be used for the remaining three schools to allocate two to the intervention group and one to the control group.

### 2.8 Data collection

#### 2.8.1 Feasibility trial

All pupils in Year 11 in the seven recruited schools will be invited to participate, with the schools in the intervention group receiving a four-week intervention, which replaces normal RSE practice, and the schools in the control group continuing with normal practice. Participating pupils will be in the study for approximately one year and asked to complete a questionnaire during an RSE lesson at baseline and at five and nine months after implementation. Schools will be encouraged to allow pupils to complete the questionnaire online but, if this is not feasible, will be permitted to use paper questionnaires. A fieldworker will be in attendance during data collection to administer the questionnaire or oversee access to the online survey. Questionnaires and envelopes (or links to the online survey) will be left for absent pupils to complete. Identical procedures will be adhered to at each of the two follow-up surveys.

#### 2.8.2 Process evaluation

The qualitative component of the study aims to assess ways in which the intervention is implemented and received in schools, and to collect information on the context of the schools, which might help explain differences in participation and implementation across schools and outcomes among pupils in the intervention and control groups. The objectives of the qualitative component are:

• To record experiences of recruiting schools, also paying attention to refusals;
• To record schools’ reactions to being assigned to control or intervention arm;
• To further test the face validity amongst pupils of the quantitative instruments (pre and post questionnaires) by conducting cognitive interviews on the topic of the questionnaire (control group only);
• To inquire into teacher trainers’ views on the training materials for teachers;
• To inquire into teachers’ views on the quality of the training session offered on the intervention (either one to one, or in a group session) and how the research team might develop a webinar and Web 2.0 support structures for future training (intervention group);
• To record the current policies on, and provision of, RSE in schools and to assess what RSE was delivered in intervention and control groups during the feasibility trial;
• To inquire into teachers’ experiences of using the intervention in the classroom and school context as well as with parents (intervention group);
• To inquire into pupils’ experiences of participating in the intervention at school and home (intervention group);
• To gain further information on implementation fidelity and teachers’, pupils’ and parents/guardians’ reactions through researcher observation.
• To inquire into parents’/guardians’ reactions to their children participating in the intervention as well as the advantages and disadvantages of their own participation; and
• To inquire into participants’ experiences of taking part in the trial.

These data will be collected through a triangulation of sources including teachers, pupil and parents/guardians and by a number of different methods, including semi-structured interviews (with 7 teachers and ‘Heads of Year’ or school principals), focus group discussions (with pupils in each participating school), and unstructured observations of a sample of RSE lessons (at least one in each participating school) as well as the parent discussion sessions (in each school in intervention group). During the process of recruiting schools, we will record any communication (by telephone in person or in writing) which indicates reasons for the school's reluctance or refusal to become involved in the study.

#### 2.8.3 Economic evaluation

The aim of the economic component is to establish whether the data collection forms we design capture resource use efficiently (i.e. collect the main cost drivers without overburdening the staff) and that the staff find them clear and easy to complete. The capture of the intervention costs will be guided by the template recommended by Ritzwoller et al. (Ritzwoller, Sukhanova, Gaglio, & Glasgow, 2009) which discriminates between trial-related costs and intervention costs (such as staff training, staff time input and teaching materials). This will include any capital costs (equipment and space), labour and other consumption costs associated with the development and delivery of the training materials. Where relevant, we will attempt to assess the variation in such costs across study sites.

A micro costing approach will be taken to estimate the costs of delivering the intervention. Where possible, costs will be gathered prospectively to provide a robust estimate of the resource use and costs associated with the delivery of the intervention. *If I Were Jack* researchers will complete timesheets and expense claim forms (including travel) on a regular basis. School staff will also document their contribution prospectively where possible. Project researchers will record other costs (e.g. training materials, venue costs, food and refreshments) in a dedicated costing spread sheet. Resource use data (e.g. staff time and consumables) will be combined with appropriate unit costs, to estimate a mean incremental cost per child to deliver the intervention, and a mean incremental cost per school. Subsequent costs associated with potential behavioural changes will also be assessed (e.g. contraception use).

### 2.9 Data analysis

Primary analyses will be on an intention-to-treat basis, using all participants in the groups they were randomised to, regardless of the intervention received.

*Participant flow and recruitment*: Summary statistics on consent, withdrawal and dropout will be collated for both trial groups. For each group, the numbers of clusters and participants randomly assigned, receiving intended intervention, completing the study protocol, and analysed for the outcomes will be presented in a CONSORT flow diagram (Schulz, Altman, & Moher, 2010).

*Baseline data*: Appropriate descriptive summaries of baseline demographic and questionnaire data for pupils and parents from the two study arms will be tabulated. Descriptive summaries will be produced for baseline data at cluster level where appropriate.

*Pupil outcomes (5 and 9 month follow-up):* Questionnaire data from pupils will be analysed using frequencies and descriptive statistics. Differences between the trial groups and broad indicators of effect size will be reported. As this is a feasibility study, it is not powered to detect significant differences and inferential analyses will not be performed. Descriptive statistics will be used to report the proportion (categorical outcomes) or mean scores (continuous outcomes) for the intervention and control groups. Reliability of psychometric instruments will be analysed through recourse to classical reliability theory involving factor analysis and calculation of Cronbach's alpha.

*Effect size estimates:* As noted, this feasibility study will measure surrogate outcomes associated with reductions in unintended pregnancy such as abstinence from sex or avoidance of unprotected sex (categorical variables). Effect sizes will be calculated from the differences in proportions between the intervention and control groups. A number of secondary outcomes are also being investigated (continuous variables). Effect sizes will be calculated from the differences in the mean scores between the intervention and control groups taking into account the standard deviation.

*Implementation feasibility:* Following transcription of all audio-recorded interviews and removal of any identifying characteristics of specific schools and individuals, the data will be analysed thematically based on the six steps proposed by Braun and Clarke (Braun & Clarke, 2006). In summary, this follows well established practice of qualitative analysis of moving from inductively derived codes from the data along with searching for data on pre-defined themes as derived from our topic guides and the literature on pupils’ teachers’ and parents’ perceptions of RSE as well as unintended pregnancy. These inductively and deductively derived codes will be analysed to form overarching themes emerging from each of the participant groups outlined above. We will use qualitative software (NVivo 10) to organise the data, and we will ensure methodological rigour by establishing credibility, transferability, dependability and confirmability using techniques suggested by Lincoln and Guba (Lincoln & Guba, 1985). In addition, following Hyde et al. (Hyde, Howlett, Brady, & Drennan, 2005), specific attention will be given to analysing the group dynamics of the focus groups as part of the overall interpretive process. In schools in the control group, special attention will be given to the specifics of analysing cognitive interviews. Feasibility of research methods will be assessed by observation in intervention schools using structured schedules, and through interviews with school staff and subjected to the same analysis.

*Cost-effectiveness:* As described above, the costs of delivering the intervention and current RSE will be captured to facilitate a cost analysis of the two strategies. The findings from the separate cost and effectiveness analyses in the feasibility study will be used to inform the design of a future economic evaluation. We will also determine the acceptability and feasibility of collecting self-report data on STIs by including questions relating to this in the questionnaire. This will help determine if we can model the cost effectiveness of the intervention in terms of reducing STIs as part of future research.

### 2.10 Ethical considerations

Ethical approval for the study has been granted by the research ethics committee of the School of Nursing & Midwifery, Queen's University Belfast in April 2014 (ref: 04.02.02.V2). We will write to parents to inform them about the research and offer them the opportunity to withdraw their child from the study. The letter will provide the full contact details of the principal investigator, research fellow and chair of the school research ethics committee and will direct parents to a short online information video about the study. Information sheets will be provided to all participants and principals, teachers and pupils will also be given a verbal overview of the research by the research fellow. Written consent will be obtained from principals, teachers, pupils and parents participating in focus groups. Confidentiality will be assured and participants will be made aware that digital recordings will be destroyed following transcription. All information pertaining to individuals and schools will be anonymous from the outset of the study.

## 3 Discussion

The need for gender-sensitive interventions to address teenage pregnancy has been highlighted as a global health need by the World Health Organisation (World Health Organisation, 2011, World Health Organisation, 2012) and others (Marsiglio, 2006, Ries and Sonenstein, 2006, Shepherd et al., 2010). We aim to initiate a process of robust scientific evaluation which will ultimately produce generalisable findings especially relating to gender-specific teenage pregnancy interventions. A successful feasibility study (with some further piloting work outside of NI) would underpin the development of a UK-wide trial. If it is acceptable and effective, the intervention has the potential to be rolled out to large numbers of boys and girls attending schools across the UK and to be of benefit to several groups of people.

Strengths of the study include the evidence-based, user-endorsed intervention and implementation protocol, which draw on scientifically supported behaviour change theories and research evidence regarding the optimal content and components of effective RSE programmes, and are enhanced by team expertise and end-user engagement in the development and evaluation process.

A limitation of the study includes the potential for implementation failure in busy school settings, and this is a key reason for beginning the evaluation with this feasibility trial. We have taken steps to optimise fidelity to implementation protocol, a factor which has been implicated in failure to demonstrate impact in previous UK-based programmes (Elliott et al., 2013, Wight, 2011). For example, in the SHARE study (a large randomised control trial of comprehensive sex education conducted in Scotland), issues arose with fidelity to the implementation protocol relating to time constraints and the low priority given to delivering the overall programme in some schools (Wight, 2011). We therefore believe that one of the strengths of the *If I Were Jack* intervention is that it is shorter than previous UK based programmes and will demand less time of the already busy RSE teacher. Furthermore, we have developed the intervention in close consultation with end users who have indicated that its content, components and implementation processes are acceptable and feasible. The intervention also includes focused one-to-one training for RSE teachers, which emphasises the importance of fidelity to protocol. We believe that these factors will help avoid the possibility that implementation failure will impede the demonstration of efficacy. On the basis of our preliminary research, and by working through a partnership model to develop this study, we believe that we are in a strong position to overcome the problems in implementing RSE in Northern Ireland and have already found the resource to be culturally sensitive and acceptable to statutory stakeholders and schools.

A further limitation includes the potential impact of cluster effects such as recruitment bias in a future phase III trial. If this feasibility trial progresses to a phase III trial, then the sample size would need to be calculated to take into account the clustered nature of the study, i.e. young people attending the same school are likely to share similarities including geography, socioeconomic status and ethnic background. The trial would therefore require a greater sample size than individually randomised trials in order to account for the cluster effect. Additionally, the resulting analysis would be analysed on two levels, the individual level and the cluster (i.e. school) level.

As noted, the extant literature includes systematic reviews which indicate the characteristics of effective school-based interventions (Kirby et al., 2006, Kirby, 2002) and recent reviews have shown the value of interactive-computer-based interventions (Bailey et al., 2010, Guse et al., 2012, Roberto et al., 2007). *If I were Jack* is a unique combination of the components of effective RSE which has been developed as a new intervention to specifically target teenage men. The study will thus add value to the broad literature on RSE by testing whether this combination of components when applied in a new intervention especially targeting men is acceptable, leading to informed research to test its effectiveness over the longer term in a larger trial. Furthermore, the specific context in which the study will take place greatly increases the possibility of demonstrating effectiveness in such a trial because, essentially, we would be assessing the impact of *If I Were Jack* by comparing intervention and control schools with (naturally occurring) low levels of RSE provision relating to unintended pregnancy. While research elsewhere in the UK reported good quality provision of RSE (Elliott et al., 2013, Henderson et al., 2007, Stephenson et al., 2003), the current provision of RSE in schools in NI is known to be low. For example, a 2010 survey revealed that a third of 11–16 year olds in NI had not learned about sexual matters and relationships during school lessons (NISRA, 2011). Thus, we have an increased chance of detecting positive change by introducing and evaluating the *If I Were Jack* resource in NI, because the contrast between the intervention and control groups will be greater than that reported elsewhere (Elliott et al., 2013, Henderson et al., 2007, Stephenson et al., 2003). Finally, as this is the first trial of an RSE intervention to include Catholic schools, demonstrating acceptability of this RSE intervention in Catholic schools will be an important contribution to RSE research more broadly.

## 4 Conclusion

This study confronts a gender bias in research on interventions targeting teenage pregnancy. It is increasingly apparent to researchers, practitioners, parents and others who work with youth that targeting teenage men is an important, yet neglected, part of addressing unintended teenage pregnancy. Gender norms in society and reproductive physiology have meant that adolescent men generally do not have the same opportunities as adolescent women to imagine an unplanned pregnancy in their lives and to think through the consequences of an unintended pregnancy for themselves (Lohan, Olivari, & Corkindale et al., 2013). Schools based RSE is a critical opportunity for opening up an understanding of relationships, sexuality and unintended pregnancy among boys and girls, but maximising this opportunity requires the development of acceptable and effective RSE resources (Downing et al., 2006, Ellis and Grey, 2004, Ingham and Hirst, 2010, Oringanje et al., 2009, Swann et al., 2003).

This article describes the protocol for a randomised feasibility trial of a schools-based teenage pregnancy RSE intervention targeting teenage men. The research is needed because there are currently no scientifically evaluated pregnancy related RSE interventions targeting young men in the UK and the research will set in motion a process of robust evaluation. In this feasibility study, key outcomes will be the quality of intervention implementation, and recruitment and retention of research participants. The study will also pilot the feasibility and acceptability of collecting demographic information and other sexual and psychosocial data relating to measuring the proposed primary and secondary outcomes in a future, larger trial, so that potential response rates can be determined, optimal data collection identified, and the costs of these data collection methods assessed.

## Funding

This protocol for a feasibility trial was produced as a result of an 10.13039/501100000269Economic and Social Research Council Grant (Grant Reference RES-189-25-0300) which funded the development of the intervention *If I were Jack*. The feasibility trial is currently funded by the UK 10.13039/501100000272National Institute for Health Research Public Health Research Programme (NIHR 10.13039/501100001921PHR 12/153/26). The views and opinions expressed in this paper are those of the authors and do not necessarily reflect those of the NIHR 10.13039/501100001921PHR Programme or the 10.13039/501100000276Department of Health.

## Conflicts of Interest

The authors report no conflicts of interest.
